# Acknowledgment to Reviewers of *Geriatrics* in 2021

**DOI:** 10.3390/geriatrics7010015

**Published:** 2022-01-27

**Authors:** Geriatrics Editorial Office

**Affiliations:** MDPI AG, St. Alban-Anlage 66, 4052 Basel, Switzerland

Rigorous peer-reviews are the basis of high-quality academic publishing. Thanks to the great efforts of our reviewers, *Geriatrics* was able to maintain its standards for the high quality of its published papers. Thanks to the contribution of our reviewers, in 2021, the median time to first decision was 16 days, and the median time to publication was 38 days. The editors would like to extend their gratitude and recognition to the following reviewers for their precious time and dedication, regardless of whether the papers they reviewed were finally published:
Agudelo, AndrésLauretani, FulvioAhrenfeldt, Linda JuelLavender, AndrewAleo, GiuseppeLee, So RyoungAlexa, Ioana DanaLeung, Doris Y. P.Alvarez Cubero, María JesúsLiang, Hai NingAmirabdollahian, FarzadLindenberg, JolandaAndo, TakafumiLiu, Guan-ZhengAndriany, MegahLjubicic, MarijaArlati, SaraLo Buglio, AurelioArmada Da Silva, Paulo Alexandre S.Lord, JanetArnal-Gómez, AnnaLoughrey, DavidÅrøen, AsbjørnLukosevicius, SauliusAzzolino, DomenicoLundman, BeritBaeyens, Jean-PierreLuzi, LivioBarbosa, AlexandreMarcinowska-Suchowierska, EwaBizzoca, DavideMarottoli, Richard A.Blanco, Juan F.Marques, AntonioBobu, Livia IonelaMars, MauriceBodomo, AdamsMartins, Anabela CorreiaBoltz, Marie P.Mauriello, FilomenaBray, JenniferMcCarthy, GeraldineBrooks, Johnell O.McElroy, Jane A.Brunetti, EnricoMedina-Perez, CarlosByeon, HaewonMelegari, GabrieleCaputo, Glenda GiorgiaMendonça, NunoCarr, SamMilgrom, PeterCassarino, MaricaMirtschink, PeterCassetta, MicheleMone, PasqualeCatic, Angela GeorgiaMourey, FranceCebrià I Iranzo, Maria Dels ÀngelsNakayama, EnriChan, Karen M. K.Nguyen, Tu N.Chen, Kun-HuiNienhaus, AlbertChen, Ping JenNijakowski, KacperChenoweth, LynnNikolic, DejanCho, Jeong-HyungNowak, Michal S.Choi, Youn-HeeOjeda-Thies, CristinaChui, KevinOng, Adrian W.Ciobanu, Adela MagdalenaPapadopoulou, SousanaCiorap, RaduParaskevas, George P.Coelho Silva, MateusPark, Myung BaeColman, RickiPárraga-Montilla, Juan A.Constantinou, Costas S.Pérez-Ros, PilarCorica, FrancescoPetzold, GuillermoCorreia, Sónia C.Pieri, MassimoCowley, AlisonPollari, MarjukkaCurto, AdriánPop, Gheorghe NicusorCybulski, MateuszPowers, James S.D’Cunha, Nathan M.Prasadh, SomasundaramDamulevičienė, GytėPurgatorio, RosaDe Craemer, MariekePutot, AlainDe Salvatore, SergioQu, NingDe Sanctis, Juan BautistaQuinlan, John FrancisDel Casale, AntonioRacette, SusanDel Riccio, MarcoRapoport, MarkDi Gennaro, LeonardoRasche, PeterDoke, MayurRavin, James G.Dubas-Jakóbczyk, KatarzynaReider, Lisa M.Dyer, AdamRequena, CarmenEconomou, AlexandraRespino, MatteoElshabrawy, HatemRibeiro, OscarEnders-Slegers, Marie JoséRobakowska, MarlenaEspinosa, EmmaRodríguez-Hurtado, DianaFábrega-Cuadros, RaquelRoe, CatherineFarmer, NicoleRojo-Pérez, FerminaFaron, Matthew L.Ronnebaum, JulieFerrari, CamillaRussell, StefanieForjuoh, Samuel NanaSalahudeen, Mohammed S.Freccero, DavidSalvi, FabioFrigstad, Svein OskarSantabarbara, JavierFrohnhofen, HelmutSantamaría-Peláez, MirianFukasawa, TakemichiSantini, SaraGabaude, CatherineSantric Milicevic, MilenaGarcía-Domínguez, LauraSarría Santamera, AntonioGarcía-Pola, María JoséScaturro, DalilaGarcía-Soidán, Jose L.Schlatterer, Daniel RobertGentil, PauloSchultze, AlexanderGermain, IsabelleSchumm, Walter R.Giannopoulos, SotiriosSekiya, HidekiGilchrist, NigelSeo, SeyoungGilissen, RenskeShah, ShamikGiménez-Llort, LydiaShimizu, AkioGlusman, GustavoShoesmith, EmilyGoettel, NicolaiSiedlecki, KarenGomula, AleksandraSilveira-Nunes, GabrielaGonzález, JerónimoSimmen, Hans-PeterGonzález-Camarena, RamónSlotved, Hans-ChristianGraf, HeikoSmith, EricGrammatopoulos, GeorgeSoares Teles, ArielGroza, RobertSoiza, Roy L.Grund, StefanStanojevic, Dragana M.Guillot, JordanStavrinou, PinelopiHan, SolaStewart, CarrieHansíková, HanaStončikaitė, IevaHaragus, HoriaStrielkowski, WadimHess, MoritzSun, JieHewitt, JonathanSuñer Soler, Maria RosaHoerr, RobertSzucs, VeronikaHogervorst, EefTavares, JoãoIosa, MarcoTchetina, Elena V.Irisawa, HiroshiTeramoto, ShinjiIshimoto, YasukoTobis, SlawomirIvan, LoredanaToledano-González, AbelIvanová, KateřinaTomás, José ManuelJang, Jong-HwaTomsone, SigneJawad, AliTroutman-Jordan, MeredithJimenez-Garcia, Jose DanielTsai, Chia-LiangJung, HeewonTsotsos, Lia E.Jung, Ki JinTumosa, NinaJürisson, MikkVaismoradi, MojtabaJurkojć, JacekVignatelli, LucaKar, AnirbanVillafaina, SantosKatayama, Pedro LourençoWard, Katherine T.Keller, LanceWawrzuta, DominikKenyon, JulianWieczorek, AnetaKim, HyangheeWimmer, MarkusKim, Hyun K.Witkowski, JacekKjeldsen, EigilWittmann, MariaKnapp, Kenneth A.Wolniak, RadosławKnauf, TomWong, Arkers Kwan ChingKonigsberg, Beau S.Wright, DavidKonrad, ThomasYoon, So-YeonKorneta, PiotrYunusa, IsmaeelKoszela, KamilZafar, Muhammad SohailKrakowski, PrzemysławZampieri, SandraKuhlmann, BeatriceZanini, MilkoKumar, SanjayZiganshina, LiliyaLabuz-Roszak, BeataZschäbitz, StefanieLara-Palomo, Inmaculada CarmenZwakhalen, Sandra M. G.Larner, Andrew J.


